# Regulation of gonadotropins by corticotropin-releasing factor and urocortin

**DOI:** 10.3389/fendo.2013.00012

**Published:** 2013-02-20

**Authors:** Kazunori Kageyama

**Affiliations:** Department of Endocrinology and Metabolism, Hirosaki University Graduate School of MedicineHirosaki, Aomori, Japan

**Keywords:** corticotropin-releasing factor, urocortin, stress, gonadotropin

## Abstract

While stress activates the hypothalamic–pituitary–adrenal (HPA) axis, it suppresses the hypothalamic–pituitary–gonadal (HPG) axis. Corticotropin-releasing factor (CRF) is a major regulatory peptide in the HPA axis during stress. Urocortin 1 (Ucn1), a member of the CRF family of peptides, has a variety of physiological functions and both CRF and Ucn1 contribute to the stress response via G protein-coupled seven transmembrane receptors. Ucn2 and Ucn3, which belong to a separate paralogous lineage from CRF, are highly selective for the CRF type 2 receptor (CRF_2_ receptor). The HPA and HPG axes interact with each other, and gonadal function and reproduction are suppressed in response to various stressors. In this review, we focus on the regulation of gonadotropins by CRF and Ucn2 in pituitary gonadotrophs and of gonadotropin-releasing hormone (GnRH) via CRF receptors in the hypothalamus. In corticotrophs, stress-induced increases in CRF stimulate Ucn2 production, which leads to the inhibition of gonadotropin secretion via the CRF_2_ receptor in the pituitary. GnRH in the hypothalamus is regulated by a variety of stress conditions. CRF is also involved in the suppression of the HPG axis, especially the GnRH pulse generator, via CRF receptors in the hypothalamus. Thus, complicated regulation of GnRH in the hypothalamus and gonadotropins in the pituitary via CRF receptors contributes to stress responses and adaptation of gonadal functions.

## INTRODUCTION

A variety of stressors have been shown to suppress gonadal function (Chand and Lovejoy, 2011). Proteins that play key roles in vertebrate reproduction include the neuropeptides gonadotropin-releasing hormone (GnRH) and kisspeptin and their receptors (Kim et al., 2012): kisspeptin stimulates GnRH release from hypothalamic GnRH neurons via Gpr54, a G protein-coupled receptor (Messager et al., 2005), while the gonadal steroid estrogen mediates its inhibitory effect on GnRH secretion by acting on kisspeptin-expressing neurons of the arcuate nucleus (Oakley et al., 2009; Ohkura et al., 2009). The expression of kisspeptin and kisspeptin receptor mRNA is downregulated by stressors including restraint, hypoglycemia, and lipopolysaccharide, which suggests that kisspeptin/kisspeptin receptor signaling plays a critical role in the transduction of stress-induced suppression of reproduction (Kinsey-Jones et al., 2009). In fact, kisspeptin–GPR54 signaling in the arcuate nucleus of the mediobasal hypothalamus is a critical neural component of the hypothalamic GnRH pulse generator (Li et al., 2009).

Gonadotropin-inhibitory hormone (GnIH), an RFamide-related peptide, can also modulate the reproduction of vertebrates (Ubuka et al., 2008). GnIH neurons interact directly with GnRH neurons, and the action of GnIH is mediated by a novel G protein-coupled receptor, Gpr147 (Ubuka et al., 2008). In mice, higher concentrations of GnIH-like substances are expressed in the hypothalamus and GnIH reduces GnRH release from the mouse hypothalamus (Bentley et al., 2010). The glucocorticoid and corticotropin-releasing factor (CRF) receptors are expressed in a large population of GnIH/RFamide-related peptide-expressing cells (Kirby et al., 2009). Glucocorticoids increase the inhibitory actions of GnIH on GnRH secretion (Kirby et al., 2009), while the regulation of GnIH via the CRF receptor remains to be determined.

Corticotropin-releasing factor activates and regulates the hypothalamic–pituitary–adrenal (HPA) axis during stress (Vale et al., 1981, 1997). Stress-induced CRF synthesis and secretion from the hypothalamic paraventricular nucleus (PVN) stimulates adrenocorticotropic hormone (ACTH) release from pituitary corticotrophs (Gillies et al., 1982; Mouri et al., 1993), which, in turn, stimulates the release of glucocorticoids from the adrenal glands (Whitnall, 1993). These glucocorticoids then moderate the stress response by inhibiting hypothalamic PVN production of CRF and pituitary production of ACTH (Whitnall, 1993). Urocortin 1 (Ucn1), a 40-amino acid peptide originally cloned from the Edinger–Westphal nucleus, is a member of the CRF family of peptides (Vaughan et al., 1995). Both CRF and Ucn1 contribute to stress responses and cardiovascular and gonadal functions via G protein-coupled seven transmembrane receptors (Vale et al., 1997; Kageyama et al., 1999a; Suda et al., 2004). CRF exhibits high affinity for CRF type 1 receptor (CRF_1_ receptor; IC_50_ = 1.6 nM) but not for CRF type 2b receptor (CRF_2b_ receptor; IC_50_ = 42 nM), while Ucn1 exhibits similar affinity for CRF_1_ receptor (IC_50_ = 0.16 nM) and CRF_2b_ receptor (IC_50_ = 0.86 nM; Jahn et al., 2004). CRF_1_ receptor is predominately expressed in the brain and pituitary gland (Chang et al., 1993; Chen et al., 1993; Vita et al., 1993; Potter et al., 1994). In the pituitary, the CRF_1_ receptor is mainly expressed by corticotrophs and is responsible for mediating the effects of hypothalamic CRF on ACTH secretion in response to stress (Wynn et al., 1985; Antoni, 1986).

Ucn2 and Ucn3 prohormones were identified in the human genome database and in mouse genomic DNA, respectively (Hsu and Hsueh, 2001; Lewis et al., 2001; Reyes et al., 2001), from which the identity and existence of endogenous peptides were predicted (Fekete and Zorrilla, 2007). Ucn2 and Ucn3 are more similar to each other than to CRF with regard to receptor binding (Kishimoto et al., 1995; Lovenberg et al., 1995a; Perrin et al., 1995; Stenzel et al., 1995). Ucn2 exhibits high affinity for CRF_2b_ receptor (IC_50_ = 0.25 nM) but low affinity for CRF_1_ receptor (IC_50_ > 350 nM; Jahn et al., 2004). Similarly, Ucn3 binds with moderate affinity to CRF_2b_ receptor (IC_50_ = 14 nM), but its specific binding to CRF_1_ receptor is not detectable (IC_50_ > 2000 nM; Jahn et al., 2004). It is hypothesized that an ancient gene duplication event is behind why Ucn1 belongs to the CRF lineage and why Ucn2 and Ucn3 represent a separate paralogous lineage (Fekete and Zorrilla, 2007).

The CRF_1_ receptor is primarily involved in stress responses and depression, while the CRF_2_ receptor is believed to mediate “stress-coping” responses in the brain, such as anxiolysis (Suda et al., 2004), because mice deficient in the CRF_2_ receptor or treated with a CRF_2_ receptor antagonist display increased anxiety-like behaviors and hypersensitive stress responses (Bale et al., 2000). Furthermore, both Ucn2 and Ucn3 act as anorexigenic neuropeptides via the CRF_2_ receptor (Fekete et al., 2011; Chao et al., 2012) and Ucn3 regulates glucose-stimulated insulin secretion and energy homeostasis (Li et al., 2007). Ucn3 signaling through the CRF_2_ receptor is also a critical molecular mediator in the ventromedial nucleus of the hypothalamus in regulating feeding and peripheral energy metabolism (Chao et al., 2012).

Corticotropin-releasing factor is involved in the suppression of the hypothalamic–pituitary–gonadal (HPG) axis (Rivier et al., 1986), especially the GnRH pulse generator in the hypothalamus (Knobil, 1992). Stress profoundly inhibits the reproductive function by suppressing the pulsatile release of GnRH and consequently luteinizing hormone (LH), at least in part via the CRF system as well as through the GABAergic system (Lin et al., 2012). Although CRF and Ucn clearly have potent effects on the HPG system, their possible roles and how they are regulated have yet to be fully determined. In this review, we focus on the regulation and the roles of Ucn2 in pituitary gonadotrophs and discuss the regulation of GnRH via CRF receptors in the hypothalamus.

## REGULATION OF GONADOTROPINS BY CRF AND Ucn2 IN THE PITUITARY 

**FIGURE 1:**
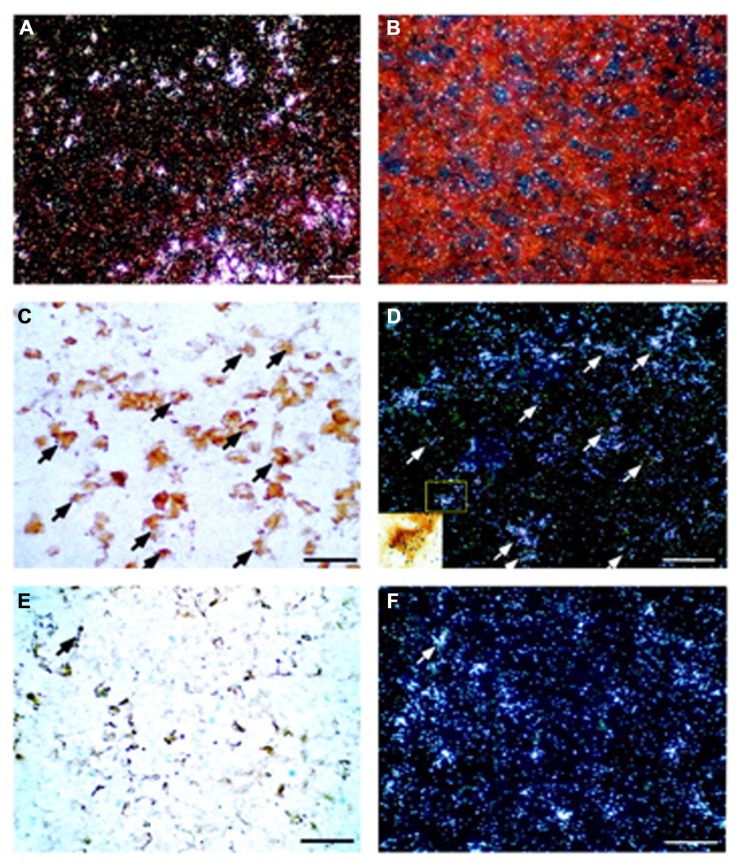
**Localization of CRF_2_ receptor mRNA in the rat anterior pituitary gland.** Representative dark-field photomicrographs showing anterior pituitary sections probed with either antisense **(A)** or sense **(B)** *in situ* hybridization probes for CRF_2_ receptor mRNA. Positive signals (indicated by *silver grain clusters*) were found only in the tissues probed with antisense probes; no signals were found in the sense control. **(C)** Bright-field photomicrograph showing LH-immunoreactive cells in the rat anterior pituitary. Gonadotrophs (visualized by the *brown* DAB precipitate) represent LH-immunoreactive cells. **(D)** Dark-field image of the same area as panel **(C)** showing CRF_2_ receptor mRNA-positive cells (*silver grain clusters*). Some of the cells that are double-labeled by immunostaining for LH and *in situ* hybridization for CRF_2_ receptor mRNA are indicated by *arrows*. *Inset*: High-power magnification of the *boxed area* of panel **(D)** to illustrate a LH-positive cell that shows a positive signal for CRF_2_ receptor mRNA (scattered *black silver grains*). **(E)** Representative bright-field photomicrograph of the anterior pituitary showing ACTH-immunoreactive cells (*brown* DAB precipitate). **(F)** Dark-field image of the same area as panel **(E)** showing CRF_2_ receptor mRNA signals (*silver grain clusters*). Only a few ACTH-immunoreactive and CRF_2_ receptor mRNA double-labeled cells (*arrow*) were observed. *Scale bar*, 50 μm. Reproduction from Kageyama et al. (2003) with permission of the publisher. Copyright 2003, The Endocrine Society.

Changes in CRF_1_ receptor expression and desensitization of the receptor in pituitary corticotrophs play a major role in modulating adaptive responses to stressors (Kageyama et al., 2006). CRF, vasopressin, lipopolysaccharides, cytokines, and glucocorticoids can negatively modulate the levels of pituitary CRF_1_ receptor mRNA (Pozzoli et al., 1996; Sakai et al., 1996; Aubry et al., 1997). However, CRF_2_ receptor mRNA is also found in the anterior pituitary and combined immunohistochemistry and *in situ* hybridization have demonstrated that CRF_2_ receptor mRNA colocalizes mainly with gonadotrophs, not corticotrophs (**Figure 1**).

RNase protection assays of anterior pituitary mRNA show that the dominant receptor type is the CRF type 2a receptor (CRF_2a_) receptor and not the CRF_2b_ receptor (Lovenberg et al., 1995a; Kageyama et al., 2003). Rat CRF_2a_ receptor, linked to various roles in the brain, is expressed primarily in several discrete brain regions, including the hypothalamus, lateral septum, and raphe nuclei (Lovenberg et al., 1995b), whereas the CRF_2b_ receptor is found predominately in peripheral tissues such as the heart, gastrointestinal tract, arterioles, and muscles (Kageyama et al., 1999b). These data suggest that the CRF_2a_ receptor in pituitary gonadotrophs is involved in the modulation of gonadotropin secretion and/or gonadal function.

Activation of the stress system could potentially influence reproduction at any level of the HPG axis (Tilbrook et al., 2002). The stress-induced decreases in LH/follicle-stimulating hormone (FSH) secretion influence gonadal functions such as sex steroidogenesis and sperm production (Demura et al., 1989; Tilbrook et al., 2002). Ucn2 is expressed mainly in corticotrophs of rat pituitary (Yamauchi et al., 2005), and its secretion and expression levels are increased by CRF and suppressed by glucocorticoids (Nemoto et al., 2007).

The CRF_2_ receptor-selective ligand Ucn2 suppresses both expression and secretion of gonadotropins in rats, while a CRF_2_ receptor antagonist increases the secretion of gonadotropins (Nemoto et al., 2009). In addition, an anti-CRF antibody blocks stress-induced increases in plasma ACTH and corticosterone, and an anti-Ucn2 antibody blocks stress-induced suppression of LH secretion without affecting stress-induced ACTH and corticosterone release (Nemoto et al., 2010). Stress-induced increases in microRNA-325-3p also suppress gonadotropin secretion (Nemoto et al., 2012). Although the presence and/or secretion of mature Ucn2 has not been determined in the pituitary or other tissues, it is possible that stress-induced increases in CRF stimulate Ucn2 in corticotrophs, which inhibits gonadotropin secretion via CRF_2_ receptors in the pituitary.

## REGULATION OF GnRH BY CRF AND Ucn VIA CRF RECEPTORS IN THE HYPOTHALAMUS

Although peripheral administration of CRF fails to affect LH secretion (D’Agata et al., 1984; Rivier and Vale, 1984), central injection of CRF inhibits secretion of gonadotropins (Rivier et al., 1986). These effects of CRF probably reflect a central mechanism that involves modulation of the activity of GnRH neurons in the hypothalamus (Petraglia et al., 1987; Li et al., 2010). Indeed, in monkeys, a CRF antagonist attenuates suppression of the GnRH pulse generator in response to hypoglycemic stress (Chen et al., 1996). Furthermore, a recent *in vivo* rat study indicated that CRF innervation of the dorsolateral bed nucleus of the stria terminalis plays a central role in stress-induced suppression of the GnRH pulse generator (Li et al., 2011).

**FIGURE 2:**
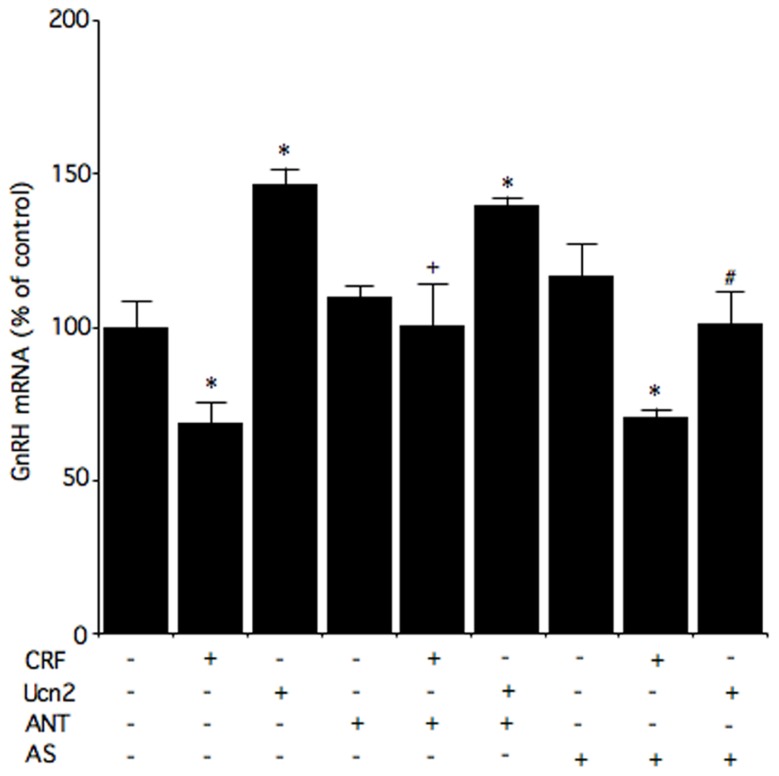
**Effects of a CRF receptor antagonist on the effects of CRF and Ucn2 on GnRH mRNA levels in N39 cells.** Cells were pre-incubated with medium containing 1 μM antalarmin (ANT), antisauvagine-30 (AS), or vehicle for 30 min, and then incubated for 6 h with medium containing 100 nM CRF, 100 nM Ucn2, or vehicle. Reproduction from Kageyama et al. (2012) with permission of the publisher. Copyright 2012, Elsevier.

Corticotropin-releasing factor also suppresses GnRH gene expression levels in murine GnRH GT1-7 cells (Kinsey-Jones et al., 2006). In fact, GT1-7 GnRH-producing cells have been used extensively in studies of the basic control mechanisms involved in GnRH neuronal function. Belsham and colleagues have managed to develop cell lines that are representative of the enormous range of cell types of the hypothalamus (Dalvi et al., 2011). N39, developed from primary mouse fetal hypothalamic culture, is one of these homologous neuronal cell lines. To further understand the possible function of Ucn and the regulation of GnRH by CRF receptors in the hypothalamus, hypothalamic N39 cells have been studied because they express both CRF_1_ and CRF_2_ receptor mRNA and protein (Kageyama et al., 2012). It has been shown in these cells that a CRF_1_ receptor antagonist, antalarmin, inhibits CRF-induced decreases in GnRH mRNA levels, which suggests that CRF decreases GnRH mRNA levels via the CRF_1_ receptor (**Figure 2**).

The CRF_2_ receptor may also be involved in the regulation of GnRH gene expression. It has been reported that CRF regulates GnRH mRNA levels via, at least in part, the CRF2 receptor in GT1-7 cells (Kinsey-Jones et al., 2006). In N39 cells, Ucn2 increases GnRH mRNA levels, and these Ucn2-induced increases in GnRH mRNA levels are blocked by the CRF_2_ receptor antagonist antisauvagine-30 (**Figure 2**). These results suggest that Ucn2 stimulates GnRH mRNA levels via the CRF_2_ receptor in hypothalamic cells. In an *in vivo* study, hypoglycemia- and lipopolysaccharide-induced suppression of LH involves activation of CRF_2_ receptor while restraint stress-induced inhibition of LH pulses involves both CRF_1_ and CRF_2_ receptors (Li et al., 2006). On the other hand, a more recent *in vivo* study showed that a CRF_1_ receptor antagonist blocks the acute stress-induced increases in gonadotropin secretion on the morning of proestrus while a CRF_2_ receptor antagonist weakly blocks the increase in FSH secretion (Traslaviña and Franci, 2012). Although GnRH production and secretion may be differentially modulated via CRF receptors under different stressors, further study will be required to elucidate the involvement of CRF receptors.

Glucocorticoids were recently shown to increase CRF_2a_ receptor expression while simultaneously inhibiting CRF_1_ receptor expression in pancreatic β cell-derived insulinoma MIN6 cells expressing glucocorticoid receptors (Huising et al., 2011). The differential effects of the glucocorticoids on the expression of these receptors in the endocrine pancreas represent a mechanism of shifting sensitivity from CRF_1_ to CRF_2_ receptor ligands (Huising et al., 2011). In the hypothalamus, glucocorticoids, released in response to stress, inhibit GnRH and gonadotropins through activation of GnIH (Kirby et al., 2009). It has yet to be determined whether glucocorticoid-induced changes in CRF and Ucn are involved in the regulation of GnRH and gonadotropins.

## RELATION BETWEEN SEXUAL DIFFERENCES AND THE CRF SYSTEM IN THE HYPOTHALAMUS

Sexual dimorphism is associated with stress sensitivity and interaction of the HPA and HPG axes (Chand and Lovejoy, 2011). Estrogens are implicated in the differing stress responses between the sexes and modulate activation of the HPA axis; females, but not males, generally have slight hypercortisolism (Magiakou et al., 1997). Estrogen replacement increases the basal levels of ACTH in ovariectomized rats (Ochedalski et al., 2007) and in postmenopausal women (Fonseca et al., 2001). Moreover, women in the midluteal phase, when both progesterone and estrogen levels are relatively high, show enhanced ACTH levels in response to a stressor (Altemus et al., 2001).

Estrogens acting centrally, including in the pituitary corticotrophs and the hypothalamus, are able to modulate the stress responses (Nakano et al., 1991), and direct estrogenic regulation of CRF gene expression has also been demonstrated in various tissues (Vamvakopoulos and Chrousos, 1993; Dibbs et al., 1997). As high levels of estrogen replacement increase the basal levels of CRF mRNA in the PVN of ovariectomized rats (Ochedalski et al., 2007), estrogen would regulate the HPA axis *in vivo* by stimulating CRF gene expression in the hypothalamus. CRF mRNA levels in the PVN are not affected by estrogen treatment in either gonadectomized estrogen receptor (ER) type β (ERβ) knockout mice or wild-type male mice (Nomura et al., 2002). Therefore, it is likely that estrogen modulates CRF gene expression in a sex-dependent manner.

Hypothalamic 4B cells show characteristics of the parvocellular neurons of the PVN because these cells express CRF, vasopressin, CRF_1_ receptor, and glucocorticoid receptors. Estrogen directly stimulates CRF gene expression in hypothalamic 4B cells (Ogura et al., 2008), suggesting that estrogen is involved in the positive regulation of CRF gene expression in the parvocellular region of the PVN *in vitro*. Neurons expressing both CRF and ERβ are found in the medial parvocellular division (Miller et al., 2004) and project to the median eminence, and CRF in parvocellular PVN neurons exerts effects on corticotroph ACTH secretion (Gillies et al., 1982; Mouri et al., 1993). Therefore, estrogen and ERβ would contribute to the enhancement of stress responses through stimulation of CRF neurons of the hypothalamus, and may constitute the basis of sexual dimorphism in the regulation of the CRF gene (Straub, 2007). In addition, estrogen also enhances CRF- and stress-induced suppression of pulsatile LH secretion (Cates et al., 2004), and upregulation of the CRF_2_ receptor may contribute to the sensitizing influence of estradiol on the CRF- and stress-induced suppression of the GnRH pulse generator (Kinsey-Jones et al., 2006).

Meanwhile, Ucn1 in the non-preganglionic Edinger–Westphal nucleus plays an important role in stress adaptation. Estrogens exert a differential transcriptional regulation of the Ucn1 gene through either ER type α (ERα) or ERβ receptors (Haeger et al., 2006). Ucn1 mRNA levels in the non-preganglionic Edinger–Westphal nucleus of male rats are much higher than those of females (Derks et al., 2010), and estrogens may contribute to stress adaptation through modulation of Ucn1 production.

## CONCLUSION

**FIGURE 3:**
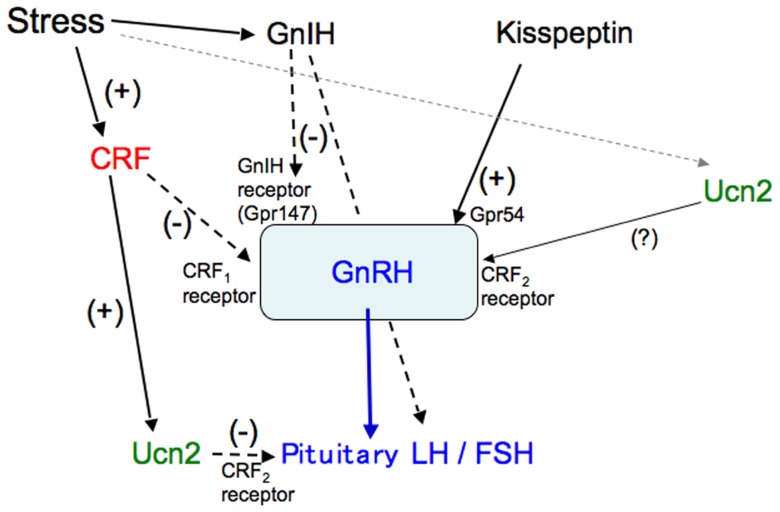
**A schematic model of the regulation of gonadotropins by CRF and Ucn.** Stress-induced increases in CRF stimulate Ucn2 in corticotrophs, which inhibits gonadotropin secretion via the CRF_2_ receptor in the pituitary. CRF inhibits the stress-induced suppression of the GnRH pulse generator and decreases GnRH mRNA levels via the CRF_1_ receptor in the hypothalamus. The CRF_2_ receptor may also be involved in the regulation of GnRH.

In summary, Ucn2, mainly produced in corticotrophs in response to CRF, acts on gonadotrophs expressing the CRF_2_ receptor and inhibits the production of gonadotropins in the pituitary (**Figure 3**). CRF is involved in the suppression of the HPG axis, especially the GnRH pulse generator in the hypothalamus, and also decreases GnRH mRNA levels via the CRF_1_ receptor (**Figure 3**). The CRF_2_ receptor may be involved in the regulation of GnRH production and secretion. GnRH production and secretion may be differentially modulated via CRF receptors in response to different stressors. Thus, complicated regulation of GnRH and gonadotropins via the CRF receptors contributes to stress responses and adaptation in gonadal functions.

## Conflict of Interest Statement

The author declares that the research was conducted in the absence of any commercial or financial relationships that could be construed as a potential conflict of interest.
